# Hepatotoxicity in hematologic malignancy and hematopoietic stem cell transplant patients receiving posaconazole or isavuconazole prophylaxis: a single-center cohort study

**DOI:** 10.1017/ash.2026.10797

**Published:** 2026-07-14

**Authors:** Samantha Steele, Courtney Nichols, Kelci Coe, Nikki Tran

**Affiliations:** 1 Pharmacy, The Ohio State University Wexner Medical Center, USA; 2 The Ohio State University Wexner Medical Center, USA

## Abstract

**Objective:**

To assess and compare the incidence, timing, and severity of hepatotoxicity associated with prophylactic isavuconazole and posaconazole in patients with hematologic malignancies or a history of hematopoietic stem cell transplant (HSCT).

**Design:**

Retrospective cohort study.

**Setting:**

Large academic tertiary-care center.

**Patients:**

Hospitalized adult patients with hematologic malignancies or a history of HSCT who received posaconazole or isavuconazole for antifungal prophylaxis between August 2015 and August 2024.

**Methods:**

The primary outcome was the incidence of hepatotoxicity, defined by elevations in aspartate aminotransferase (AST), alanine aminotransferase (ALT), alkaline phosphatase (ALP), or bilirubin. Secondary outcomes included time to hepatotoxicity, severity grading per Common Terminology Criteria for Adverse Events (CTCAE) v5.0., and causality assessment using the Naranjo Adverse Drug Reaction Probability Scale.

**Results:**

A total of 154 patients were included (100 receiving posaconazole, 54 receiving isavuconazole). Hepatotoxicity occurred in 17% of the isavuconazole group and 7% of the posaconazole group (*P* = .06), with all events classified as mild. The median time to hepatotoxicity was longer for isavuconazole (96 vs. 15 days, *P* = .16). Survival analysis showed no significant difference in time to hepatotoxicity between groups (*P* = .07). Most hepatotoxic events were classified as “possible” according to the Naranjo Scale.

**Conclusions:**

In this retrospective analysis, no statistically significant difference in hepatotoxicity incidence was observed between isavuconazole and posaconazole for prophylaxis. Larger prospective studies are needed to confirm these findings and further define the hepatic safety profiles of these antifungal agents.

## Introduction

Patients with hematologic malignancies and those undergoing hematopoietic stem cell transplantation (HSCT) are at increased risk for invasive mold infections and frequently require prophylactic antifungal therapy. These populations are also particularly vulnerable to drug-induced hepatotoxicity due to factors such as underlying liver dysfunction and prolonged treatment durations.^1,2^

Isavuconazole and posaconazole are broad-spectrum, second-generation triazoles commonly used for prophylaxis and treatment of invasive fungal infections in these high-risk settings.^1,3^ Both agents are recommended for the prevention of invasive fungal disease and have demonstrated efficacy in this population.^1,3,4^ However, hepatotoxicity is a known adverse effect of both drugs, with liver enzyme abnormalities reported in clinical trials involving comparisons with voriconazole.^5–7^ Despite their frequent use and similar clinical indications, no studies have directly compared the hepatotoxicity profiles of isavuconazole and posaconazole. Given their overlapping roles in antifungal prophylaxis and the potential need to switch between agents due to adverse effects, a direct comparison of hepatotoxicity is crucial to the clinical literature. This study aims to evaluate and compare the incidence, timing, and severity of hepatotoxicity associated with prophylactic isavuconazole and posaconazole in patients with hematologic malignancies or a history of HSCT.

## Methods

### Study design

This was a single-center, retrospective, cohort study conducted at The Ohio State University Wexner Medical Center on patients with hematologic malignancies or a history of HSCT who received posaconazole or isavuconazole for antifungal prophylaxis between August 1, 2015, to August 1, 2024. Data were collected via the electronic medical record, Integrated Health Information System (IHIS).

Eligible patients were adults aged 18–89 years who received azole prophylaxis while inpatient and completed at least 7 days of therapy. Patients were required to have baseline liver function tests (LFTs) obtained within one month prior to azole initiation and at least one follow-up LFT panel after azole initiation during the study period. Patients were excluded if they met any of the following criteria: prisoner status, pregnancy, receipt of an alternative azole therapy for 5 or more days within the past two weeks, underlying liver disease, or clinical conditions associated with abnormal LFTs or increased risk of hepatotoxicity. Specific conditions for exclusion included a history of any viral hepatitis, autoimmune hepatitis, end-organ liver disease due to viral infections (including specifically cytomegalovirus, herpes simplex virus, or adenovirus), nonalcoholic fatty liver disease, alcoholic liver disease, cirrhosis, history of liver transplantation, veno-occlusive disease or liver graft-versus-host disease after HSCT, primary or secondary hepatic tumors, or elevated baseline LFTs (AST or ALT above 3 times upper limit of normal [ULN]).^8^ Patients meeting the inclusion criteria were categorized into two groups based on their antifungal prophylaxis: (1) posaconazole and (2) isavuconazole.

### Data collection

Baseline demographic and clinical characteristics collected are summarized in Table 1.

**Table 1.** Baseline and clinical characteristics Table 1 long description.

Characteristics	Posaconazole (n = 100)	Isavuconazole (n = 54)	*P* value
Age, years, median (IQR)	57.5 (46–64)	62 (56–68)	0.02
Male, sex, n (%)	61 (61)	32 (59)	0.83
**Ethnicity, n (%)**			0.92
African American	4 (4)	2 (4)	
Caucasian	91 (91)	50 (93)	
Hispanic	0 (0)	0 (0)	
Other	5 (5)	2 (4)	
**Underlying disease state, n (%)**			0.001
Acute myeloid leukemia	75 (75)	25 (46)	
Acute lymphoblastic leukemia	0 (0)	8 (15)	
Myelodysplastic syndrome	7 (7)	6 (11)	
Chronic myeloid leukemia	2 (2)	2 (4)	
Non-Hodgkin’s lymphoma	0 (0)	1 (2)	
Autologous HSCT	1 (1)	0 (0)	
Allogenic HSCT	4 (4)	5 (9)	
CAR T-cell therapy	2 (2)	0 (0)	
Other hematologic malignancy	9 (9)	7 (13)	
Duration of azole therapy, days, median (IQR)	21 (16.5–25)	102.5 (28–181)	0.01

CAR, chimeric antigen receptor; IQR, interquartile range; HSCT, hematopoietic stem cell transplant.

If hepatotoxicity occurred, its severity, time from therapy initiation to onset, and the likelihood of an adverse drug reaction based on the Naranjo Adverse Drug Reaction Probability Scale were recorded. For patients who met the hepatotoxicity end point, elevations in bilirubin, ALT, AST, ALP, and INR were categorized according to CTCAE v5.0 criteria.^9,10^ Patients were followed from azole initiation until azole discontinuation, development of hepatotoxicity, loss to follow-up, or death.

### Outcomes

The primary outcome was the incidence of hepatotoxicity, defined as any of the following: AST > 3 times ULN, ALT > 3 times ULN, ALP > 2 times ULN, or total serum bilirubin >2.5 mg/dL accompanied by elevated AST, ALT, or ALP.

Secondary outcomes included time to hepatotoxicity, severity of hepatotoxicity, grading of increased bilirubin, ALT, AST, ALP, and international normalized ratio (INR) according to CTCAE v5.0 definitions, and classification of adverse drug reaction probability with the Naranjo Adverse Drug Reaction Probability Scale in those with hepatotoxicity.^9,10^

### Statistical analysis

Descriptive statistics were used to compare baseline characteristics between groups. Continuous variables were analyzed using the Wilcoxon rank-sum test or Student’s t-test, and categorical variables using the χ^2^ or Fisher’s exact test. Time-to-event analysis was performed for hepatotoxicity. Statistical significance was defined as *P* < .05.

## Results

A total of 1,765 patients who received either posaconazole or isavuconazole were initially identified with 100 and 54 patients included in the final posaconazole and isavuconazole cohorts, respectively, after exclusions (Appendix A). The most common reasons for exclusion were less than 7 days of azole therapy, non-hematology/oncology diagnoses, and prior azole use. Patients in the isavuconazole group were older and had a longer median duration of azole therapy compared with the posaconazole group (*P* = .01). Acute myeloid leukemia (AML) was the predominant underlying condition in both groups (Table 1).

**Figure 1.**
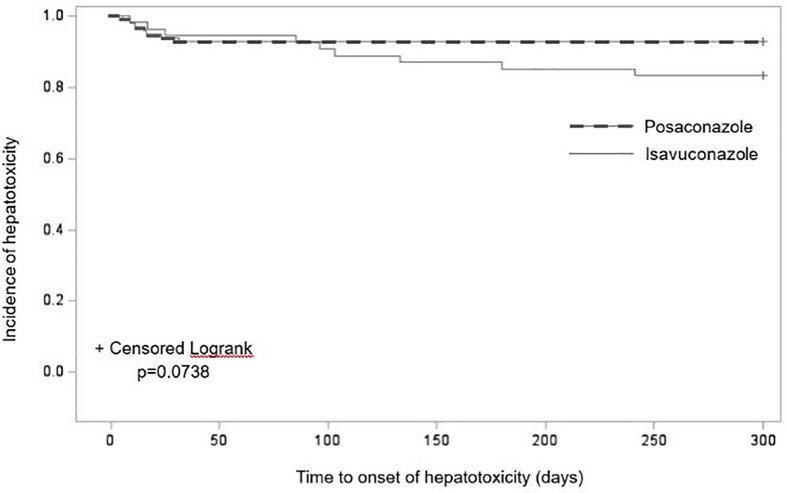
Survival probability curve for time to hepatotoxicity.

The primary outcome of hepatotoxicity occurred more frequently in the isavuconazole group (17%) compared to the posaconazole group (7%), though this difference did not reach statistical significance (*P* = .06). Notably, all cases of hepatotoxicity were classified as mild in severity (Table 2). The median time to onset of hepatotoxicity was longer among patients receiving isavuconazole (96 d) compared to those on posaconazole (15 d); however, this difference was also not statistically significant on univariate analysis (*P* = .16) (Table 2). Similarly, a survival analysis evaluating time to hepatotoxicity demonstrated no significant difference between the two groups (*P* = .07) as shown on Figure 1. In the posaconazole group, hepatotoxicity occurred on days 5, 9, 11, 15, 16, 23, and 31, with all cases arising within the first 31 days of therapy. Isavuconazole-related hepatotoxicity was more widely distributed, occurring on days 9, 17, 25, 85, 96, 103, 133, 180, and 241.

**Table 2.** Primary and secondary outcomes Table 2 long description.

	Posaconazole (n = 100)	Isavuconazole (n =54)	*P* value
Presence of hepatotoxicity, n (%)	7 (7)	9 (17)	0.06
Time to hepatotoxicity, days, median (IQR)	15 (9–23)	96 (25–133)	0.16
**Severity of hepatotoxicity, n (%)**			0.06
Mild	7 (7)	9 (17)	
Moderate	0 (0)	0 (0)	
Moderate to severe	0 (0)	0 (0)	
Severe	0 (0)	0 (0)	
**Naranjo adverse drug reaction probability scale scores, n (%)**			0.10
Score ≤ 0 (Doubtful)	93 (93)	45 (83)	
Score 1–4 (Possible)	6 (6)	9 (17)	
Score 5–8 (Probable)	1 (1)	0 (0)	
Score ≥ 9 (Definite)	0 (0)	0 (0)	
**CTCAE v5.0 Grading of hepatotoxicity, n (%)**			
Increased bilirubin			
Grade 1	1 (1)	1 (2)	1
Increased alanine aminotransferase (ALT)			0.46
Grade 1	2 (2)	1 (2)	
Grade 2	2 (2)	5 (9)	
Grade 3	2 (2)	3 (6)	
Increased aspartate aminotransferase (AST)			0.07
Grade 1	1 (1)	6 (11)	
Grade 2	5 (5)	1 (2)	
Grade 3	1 (1)	1 (2)	
Increased alkaline phosphatase (ALP)			0.68
Grade 1	1 (1)	1 (2)	
Grade 2	1 (1)	3 (6)	
Increased international normalized ratio (INR)			
Grade 1	1 (1)	1 (2)	1

CTCAE, Common Terminology Criteria for Adverse Events; IQR, interquartile range.

Causality assessment using the Naranjo algorithm determined that all hepatotoxicity events were rated as “possible” in relation to azole therapy, except for one patient in the posaconazole group, whose event was classified as “probable.” Laboratory parameters including bilirubin, ALT, AST, ALP, and INR were graded according to CTCAE v5.0 criteria and were comparable between the two treatment groups (Table 2).

## Discussion

In this single-center, retrospective cohort study of patients with hematologic malignancies or a history of HSCT, we observed a numerically higher incidence of hepatotoxicity among patients receiving isavuconazole prophylaxis compared to those receiving posaconazole (17% vs 7%), although the difference did not reach statistical significance. All hepatotoxicity events in both groups were mild in severity and primarily occurred within the first month for posaconazole, whereas hepatotoxicity associated with isavuconazole was more delayed and distributed over a broader time frame. At our institution, selection of antifungal prophylaxis is not routinely based on baseline hepatic function. Instead, antifungal agent selection is primarily influenced by factors such as compatibility with clinical trial protocols, baseline QTc interval considerations, drug-drug interaction profiles, and insurance coverage or formulary access. Therefore, baseline liver function is unlikely to represent a significant source of selection bias. Additionally, per institutional protocol, all patients receiving azole antifungal prophylaxis undergo routine weekly liver function monitoring. The observed differences in hepatotoxicity between the groups may be partly attributed to variations in treatment duration and clinical context. Posaconazole was most often used as standard prophylaxis while isavuconazole was frequently employed in patients with a history of fungal infection. This difference in treatment duration was reflected in the significantly longer median therapy duration in the isavuconazole group, potentially increasing cumulative drug exposure and the associated risk of hepatotoxicity. Additionally, the longer median time to hepatotoxicity with isavuconazole (96 vs 15 d, *P* = .16), though not statistically significant, further suggests that prolonged exposure could contribute to the delayed onset of hepatotoxicity. The delay in hepatotoxicity with isavuconazole may suggest a different time course for the development of liver injury compared to posaconazole, warranting further investigation.

Our study is the first to directly compare the hepatotoxicity profiles of isavuconazole and posaconazole in a cohort of high-risk patients, addressing a gap in the existing literature. While our findings align with certain trends observed in previously published studies, they also highlight some key distinctions. A posthoc analysis of the SECURE trial in 2016 assessed the hepatic safety profiles of isavuconazole and voriconazole in patients with invasive mold infections, including those with and without allogeneic HSCT. This analysis found that isavuconazole had a more favorable hepatic safety profile compared to voriconazole, with fewer instances of ALT or AST elevations exceeding three times the upper limit of normal.^5^ Similarly, a phase 3 non-inferiority trial comparing posaconazole and voriconazole did not identify significant differences in hepatotoxicity, as measured by ALT, AST, and bilirubin levels.^6^ A 2019 retrospective study examining the tolerability of isavuconazole in leukemia patients who had discontinued posaconazole due to hepatotoxicity reported that none of the 23 patients experienced toxicity upon switching to isavuconazole.^7^ These findings suggest that isavuconazole may offer improved tolerability in certain high-risk populations, though direct comparisons of isavuconazole and posaconazole with respect to hepatotoxicity are lacking. Additionally, prior research suggests that hepatotoxicity with one azole does not necessarily predict intolerance to other azoles, and switching agents may facilitate recovery from liver injury.

Strengths of our study include rigorous exclusion criteria to minimize baseline hepatic confounders, structured assessment tools for hepatotoxicity severity and causality, and a focus on a clinically relevant population susceptible to drug-induced liver injury due to underlying conditions and polypharmacy. Several limitations should be considered when interpreting these findings. The retrospective design is subject to residual confounding and cannot establish causality. Longer isavuconazole exposure may have increased opportunities for hepatotoxicity detection due to prolonged monitoring. Additionally, concomitant hepatotoxic chemotherapy agents were not individually controlled for; however, causality assessments were performed using the Naranjo Adverse Drug Reaction Probability Scale to help evaluate the likelihood that events were attributable to azole therapy rather than other treatments, including chemotherapy. Posaconazole therapeutic drug monitoring was not routinely available for all patients during the study period, limiting assessment of the relationship between drug exposure and hepatotoxicity.

In conclusion, no statistically significant difference in the incidence of hepatotoxicity was observed between isavuconazole and posaconazole used for prophylaxis. While these findings offer valuable insights into hepatotoxicity risk in high-risk patient populations, larger prospective studies are needed to better elucidate these results and clarify the long-term safety profiles of these antifungal agents.

## Data Availability

The data underlying this article are available in the article.

## Table 1. Long description

A table comparing baseline and clinical characteristics of two groups. The table has 12 rows and 4 columns. Column headers are Characteristics, Posaconazole (n = 100), Isavuconazole (n = 54), and P value. Row labels include Age, Male sex, Ethnicity, Underlying disease state, and Duration of azole therapy. Row 1: Age, years, median (IQR), Posaconazole 57.5 (46–64), Isavuconazole 62 (56–68), P value 0.02. Row 2: Male, sex, n (%), Posaconazole 61 (61), Isavuconazole 32 (59), P value 0.83. Row 3: Ethnicity, n (%), African American, Posaconazole 4 (4), Isavuconazole 2 (4), P value 0.92. Row 4: Ethnicity, n (%), Caucasian, Posaconazole 91 (91), Isavuconazole 50 (93), P value 0.92. Row 5: Ethnicity, n (%), Hispanic, Posaconazole 0 (0), Isavuconazole 0 (0), P value 0.92. Row 6: Ethnicity, n (%), Other, Posaconazole 5 (5), Isavuconazole 2 (4), P value 0.92. Row 7: Underlying disease state, n (%), Acute myeloid leukemia, Posaconazole 75 (75), Isavuconazole 25 (46), P value 0.001. Row 8: Underlying disease state, n (%), Acute lymphoblastic leukemia, Posaconazole 0 (0), Isavuconazole 8 (15), P value 0.001. Row 9: Underlying disease state, n (%), Myelodysplastic syndrome, Posaconazole 7 (7), Isavuconazole 6 (11), P value 0.001. Row 10: Underlying disease state, n (%), Chronic myeloid leukemia, Posaconazole 2 (2), Isavuconazole 2 (4), P value 0.001. Row 11: Underlying disease state, n (%), Non-Hodgkin’s lymphoma, Posaconazole 0 (0), Isavuconazole 1 (2), P value 0.001. Row 12: Duration of azole therapy, days, median (IQR), Posaconazole 21 (16.5–25), Isavuconazole 102.5 (28–181), P value 0.01.

Navigate back to Table 1..

## Table 2. Long description

A table comparing hepatotoxicity outcomes between posaconazole and isavuconazole groups. The table has 13 rows and 5 columns. Column headers are: Posaconazole (n = 100), Isavuconazole (n = 54), P value. Row labels include: Presence of hepatotoxicity, n (%), Time to hepatotoxicity, days, median (IQR), Severity of hepatotoxicity, n (%), Naranjo adverse drug reaction probability scale scores, n (%), CTCAE v5.0 Grading of hepatotoxicity, n (%). Row 1: Presence of hepatotoxicity, n (%), Posaconazole, 7 (7); Isavuconazole, 9 (17); P value, 0.06. Row 2: Time to hepatotoxicity, days, median (IQR), Posaconazole, 15 (9–23); Isavuconazole, 96 (25–133); P value, 0.16. Row 3: Severity of hepatotoxicity, n (%), Posaconazole, 7 (7); Isavuconazole, 9 (17%); P value, 0.06. Row 4: Naranjo adverse drug reaction probability scale scores, n (%), Score ≤ 0 (Doubtful), Posaconazole, 93 (93); Isavuconazole, 45 (83); P value, 0.10. Row 5: Naranjo adverse drug reaction probability scale scores, n (%), Score 1–4 (Possible), Posaconazole, 6 (6); Isavuconazole, 9 (17); P value, 0.10. Row 6: Naranjo adverse drug reaction probability scale scores, n (%), Score 5–8 (Probable), Posaconazole, 1 (1); Isavuconazole, 0 (0); P value, 0.10. Row 7: Naranjo adverse drug reaction probability scale scores, n (%), Score ≥ 9 (Definite), Posaconazole, 0 (0); Isavuconazole, 0 (0); P value, 0.10. Row 8: CTCAE v5.0 Grading of hepatotoxicity, n (%), Increased bilirubin, Grade 1, Posaconazole, 1 (1); Isavuconazole, 1 (2); P value, 1. Row 9: CTCAE v5.0 Grading of hepatotoxicity, n (%), Increased alanine aminotransferase (ALT), Grade 1, Posaconazole, 2 (2); Isavuconazole, 1 (2); P value, 0.46. Row 10: CTCAE v5.0 Grading of hepatotoxicity, n (%), Increased alanine aminotransferase (ALT), Grade 2, Posaconazole, 2 (2); Isavuconazole, 5 (9); P value, 0.46. Row 11: CTCAE v5.0 Grading of hepatotoxicity, n (%), Increased alanine aminotransferase (ALT), Grade 3, Posaconazole, 2 (2); Isavuconazole, 3 (6); P value, 0.46. Row 12: CTCAE v5.0 Grading of hepatotoxicity, n (%), Increased aspartate aminotransferase (AST), Grade 1, Posaconazole, 1 (1); Isavuconazole, 6 (11); P value, 0.07. Row 13: CTCAE v5.0 Grading of hepatotoxicity, n (%), Increased aspartate aminotransferase (AST), Grade 2, Posaconazole, 5 (5); Isavuconazole, 1 (2); P value, 0.07. Row 14: CTCAE v5.0 Grading of hepatotoxicity, n (%), Increased aspartate aminotransferase (AST), Grade 3, Posaconazole, 1 (1); Isavuconazole, 1 (2); P value, 0.07. Row 15: CTCAE v5.0 Grading of hepatotoxicity, n (%), Increased alkaline phosphatase (ALP), Grade 1, Posaconazole, 1 (1); Isavuconazole, 1 (2); P value, 0.68. Row 16: CTCAE v5.0 Grading of hepatotoxicity, n (%), Increased alkaline phosphatase (ALP), Grade 2, Posaconazole, 1 (1); Isavuconazole, 3 (6); P value, 0.68. Row 17: CTCAE v5.0 Grading of hepatotoxicity, n (%), Increased international normalized ratio (INR), Grade 1, Posaconazole, 1 (1); Isavuconazole, 1 (2); P value, 1.

Navigate back to Table 2..
